# Hernie sus ombilicale étranglée à contenu hépatique: à propos d´un cas

**DOI:** 10.11604/pamj.2021.39.157.30440

**Published:** 2021-06-29

**Authors:** Amal Hajri, Nassima Fakhiri, Mounir Bouali, Abdelilah Elbakouri, Khalid Elhattabi, Fatimazahra Bensardi, Abdelaziz Fadil

**Affiliations:** 1 Département des Urgences de la Chirurgie Viscérale, Université Hassan II de Casablanca, CHU Ibn Rochd, Casablanca, Maroc

**Keywords:** Hernie hépatique, fonction hépatique, TDM abdominale, à propos d’un cas, Herniation of liver segments, liver function, abdominal CT scan, case report

## Abstract

La hernie du foie à travers un défet de la paroi abdominale antérieure est rare. A notre connaissance, seuls trois cas ont été décrits dans la littérature. Un homme âgé de 84 ans s'est présenté aux urgences pour un tableau d'hernie de la ligne blanche étranglée avec un aspect terne à la percussion et la protrusion d'une masse, sensible et ferme. Les résultats des tests de laboratoire étaient normaux. Une tomodensitométrie (TDM) abdominale a montré une hernie de la ligne blanche à contenu hépatique avec un aspect de cholécystite lithiasique. Le patient bénéficiait d´une cholécystectomie rétrograde avec drainage sous hépatique par drain de Redon et Cure de la hernie sus ombilicale par paletot associe à un drainage sous cutané par 2 drains de Redon aspiratifs. Les suites post opératoires était simples et le patient était déclaré sortant au 2^e^ jour post opératoire. Six mois après l'opération, notre patient reste en bonne santé. La hernie de la paroi abdominale à contenu hépatique est rare, les signes cliniques sont souvent pauvres. La TDM abdominale est un examen indispensable pour évaluer la viabilité du parenchyme hépatique la prise en charge est étudiée cas par cas soit un traitement chirurgical ou un traitement non chirurgical. La hernie hépatique est une situation rare, peu de cas dans la littérature ont été rapportés, les signes cliniques sont souvent pauvres. La tomodensitométrie est un examen indispensable pour évaluer la viabilité du parenchyme hépatique.

## Abstract (en)

Herniation of liver segments through defects in the anterior abdominal wall is rare. To our knowledge, only three cases have been described in the literature. This study reports the case of a 84-year-old man presenting to the emergency department with strangulated hernia of the linea alba showing dullness to percussion with protrusion of a sensitive and firm mass. Laboratory test results were normal. Abdominal CT scan showed herniation of liver segment through the linea alba with calculous cholecystitis. The patient underwent retrograde cholecystectomy with subhepatic Redon drain and repair of the herniation by overlapping suture, associated with two subcutaneous vacuum suction Redon drains. The post-operative suite was simple and the patient was discharged on the second post-operative day. Six months after surgery, our patient was healthy. Herniation of liver segments through defects in the anterior abdominal wall is rare. Patients usually show little evidence if clinical signs. Abdominal CT scan is essential to assess the viability of parenchymal liver cells. Patients´ management is studied on a case-by-case basis, it can be surgical or non-surgical. Herniation of liver segments is rare; few cases have been reported in the literature and, in these cases, patients showed little evidence of clinical signs. CT scan is essential to assess the viability of parenchymal liver cells.

## Introduction

La hernie du foie est une entité rare. Elle a été décrite chez des patients présentant une omphalocèle, une hernie diaphragmatique congénitale ou une rupture diaphragmatique due à un traumatisme [1,2]. A notre connaissance, seuls trois cas de hernie hépatique à travers une hernie antérieure de la paroi abdominale ont été décrits [3,4] nous rapportons le cas d'un patient âgé de 84 ans sans antécédents pathologiques particuliers admis pour une hernie de la ligne blanche abdominale sus ombilicale étranglée à contenu hépatique.

## Patient et observation

**Information du patient:** il s´agit d´un patient âgé de 84 ans sans antécédents pathologiques particuliers présentant 2 ans auparavant une tuméfaction en regard de la ligne blanche sus ombilicale qui était réductible impulsive à la toux sans signes inflammatoires en regard, devenant 24H avant son admission au urgences douloureuse sans trouble de transit, ni hémorragie digestive extériorisée, le tout évoluant dans un contexte d´apyrexie et de conservation de l´état général.

**Figure 1:**
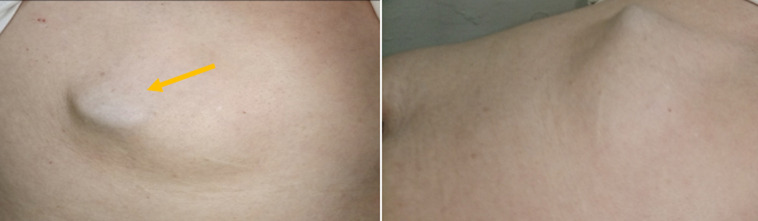
hernie sus ombilicale étranglée

**Résultats cliniques:** l´examen clinique objectivait un patient conscient stable sur le plan hémodynamique et respiratoire avec une hernie sus ombilicale étranglée sans signes inflammatoires en regard dont le contenu est de consistance ferme faisant évoquer un contenu hépatique (Figure 1).

**Figure 2:**
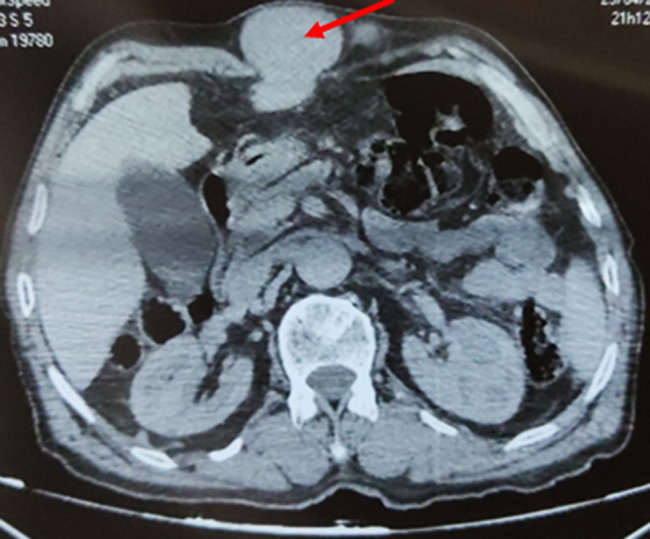
coupe scanographique transversale montrant la hernie du lobe gauche du foie à travers le défect pariétale (flèche)

**Démarche diagnostique:** la tomodensitométrie (TDM) abdominale objectivait une hernie de la ligne blanche sous xiphoïdienne dont le sac mesurait 81x39x39mm et le collet 32mm de diamètre contenant le segment III du foie présentant un défaut de rehaussement, contenant également une partie du grand épiploon associé à un épanchement liquidien. Par ailleurs, le scanner avait objectivé également une vésicule biliaire lithiasique distendue, mesurant 102 mm de grand axe et une VBP mesurée à 9 mm de diamètre sans obstacle décelable (Figure 2). Le bilan biologique montrait une hémoglobine: 13,5g/dl, globules blancs: 6710 /mm^3^, plaquettes: 186000/mm^3^, le bilan d´hémostase TP: 80%. Une fonction rénale normale urée: 0,29 g/l, créatininémie: 7,2mg/l, la fonction hépatique normale ALAT: 56 UI/l, ASAT: 69UI/l, Gamma GT: 12UI/l, phosphatase alcaline: 100UI/l, bilirubine totale: 4,4 mg/l, bilirubine conjuguée: 1,9 mg/l, bilirubine non conjuguée: 2,5mg/l, protéine C-réactive (CRP): 6 mg/l.

**Figure 3:**
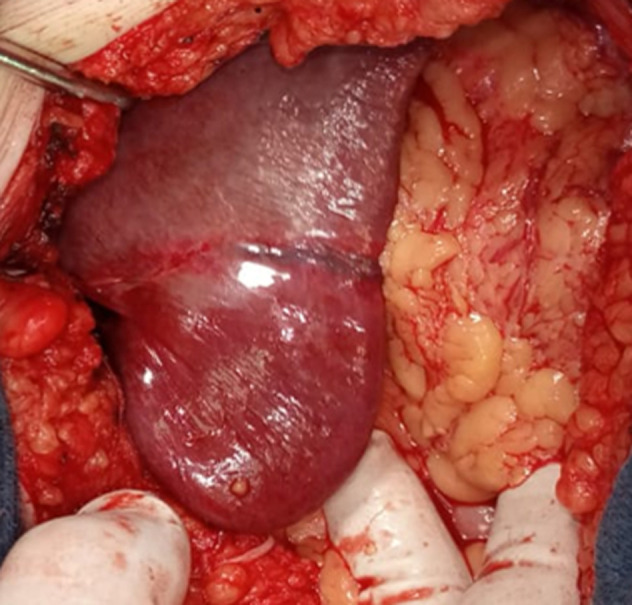
image per opératoire montrant le contenu hépatique et épiploïque de la hernie

**Figure 4:**
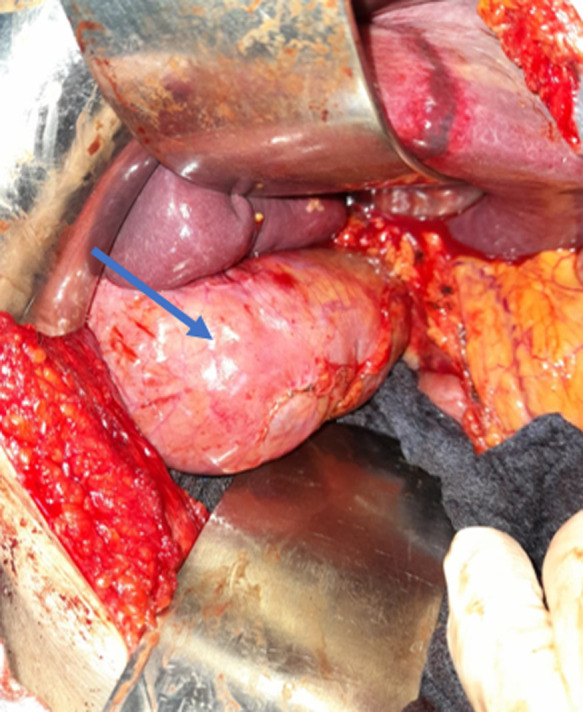
image per opératoire montrant la vésicule biliaire distendue à paroi épaissie (flèche)

**Figure 5:**
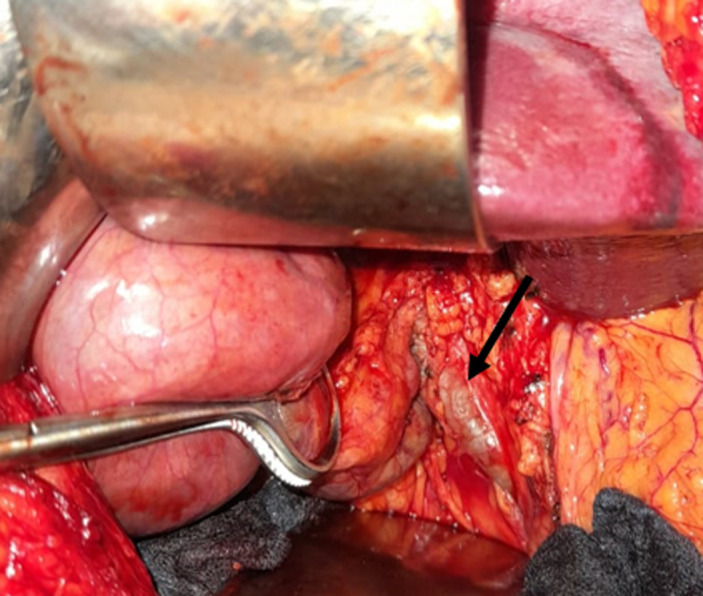
image per opératoire montrant la voie biliaire principale (flèche)

**Intervention thérapeutique et suivi:** le patient avait bénéficié d´une cholécystectomie rétrograde avec un drainage sous hépatique par drain de Redon et une cure de la hernie sus ombilicale par paletot associé à un drainage sous cutané par 2 drains de Redon aspiratifs. L´exploration avait objectivé la présence d´une hernie sus ombilicale à contenu épiploïque et le segment III du foie viable (Figure 3) dont le collet mesure 6cm et une vésicule biliaire distendue à 10cm à paroi épaissie lithiasique (Figure 4) et la voie biliaire était fine mesurant environ 9mm de diamètre (Figure 5). Les suites postopératoires étaient simples et le malade déclaré sortant à j2 post opératoire. Le recul était de six mois sans récidive.

## Discussion

La hernie a contenu hépatique à travers un défect de la paroi abdominale antérieure est rare [3, 4]. Les femmes semblent être plus susceptibles de souffrir de hernies hépatiques [5] que les hommes. La rareté de cette pathologie rend presque impossible la description spécifique des facteurs de risque prédisposants à une hernie hépatique [6]. D'après la littérature disponible, les symptômes les plus fréquents sont les douleurs abdominales, les nausées et les vomissements. Le tableau clinique est souvent non aigu sans péritonite. Cela est dû probablement au fait que le collet de la hernie est large et à l´absence d'étranglement [6]. De plus, dans la plupart des cas, il y a des bords d'hernie clairement définis qui sont facilement palpables à l´examen comme le cas de notre patient ou le contenu hépatique était suspecté à l´examen clinique. En termes d'investigation, la tomodensitométrie améliorée par le contraste semble être très sensible dans la hernie hépatique afin d´évaluer toute atteinte vasculaire ou du parenchyme hépatique [7].

La prise en charge dépend de chaque patient. Le traitement des hernies hépatiques doit être soigneusement étudié par une équipe multidisciplinaire au cas par cas. Et peut inclure un traitement non chirurgical ou chirurgical. Les patients asymptomatiques dont les tests de la fonction hépatique sont normaux ou qui présentent des comorbidités plus importantes peuvent être envisagés pour une prise en charge non chirurgicale. Une prise en charge chirurgicale doit être envisagée en cas d´hernie hépatique avec strangulation provoquant un dérèglement de la fonction hépatique et une insuffisance hépatique [7] notre patient avait bénéficié d´une prise en charge chirurgicale. Warbrick-Smith *et al*. [8] avaient rapporté le cas d´un homme âgé de 81 ans présentant une hernie hépatique de segment II/III via un défect de la paroi abdominale et une cholécystite de fortuite. Traitée de manière conservatrice. En revanche, Eken *et al*. [9] avaient rapporté le cas d'une femme de 77 ans nécessitant une fermeture primaire du défect pariétal et un patch onlay pour l´incarcération d´un segment II/III du foie dans une éventration post opératoire, avec une insuffisance hépatique aiguë.

**Consentement:** conformément aux normes internationales ou universitaires, le consentement écrit du patient a été recueilli et conservé par les auteurs.

## Conclusion

La hernie hépatique est une situation rare, peu de cas dans la littérature ont été rapportés, les signes cliniques sont souvent pauvres mais les caractéristiques sémiologiques peuvent être évocatrices. La tomodensitométrie est un examen indispensable pour évaluer la viabilité du parenchyme hépatique. Malgré sa rareté elle doit être connue par les chirurgiens pour une meilleure prise en charge.
